# Significant Associations of Neurological Complications of Herpes Zoster With Stroke in Rheumatoid Arthritis Patients

**DOI:** 10.1161/JAHA.117.006304

**Published:** 2017-07-19

**Authors:** Tsai‐Ling Liao, Ching‐Heng Lin, Hsin‐Hua Chen, Yi‐Ming Chen, Che‐Chen Lin, Der‐Yuan Chen

**Affiliations:** ^1^ Department of Medical Research Taichung Veterans General Hospital Taichung Taiwan; ^2^ Department of Internal Medicine and Medical Education Taichung Veterans General Hospital Taichung Taiwan; ^3^ Division of Allergy, Immunology and Rheumatology Taichung Veterans General Hospital Taichung Taiwan; ^4^ Rong Hsing Research Center for Translational Medicine National Chung Hsing University Taichung Taiwan; ^5^ National Taipei University of Nursing and Health Science Taipei Taiwan; ^6^ Faculty of Medicine National Yang Ming University Taipei Taiwan

**Keywords:** herpes zoster, immunosuppressive therapy, infectious disease, nervous system, neurological complications, rheumatoid arthritis, stroke, Epidemiology, Risk Factors

## Abstract

**Background:**

Accumulating evidence suggests an increased risk of stroke after herpes zoster (HZ). This risk is elevated in immunocompromised patients. The incidence of HZ in Asia is higher than in Western countries. However, the epidemiology of HZ and HZ‐related stroke among rheumatoid arthritis (RA) patients in Asia remains unclear.

**Methods and Results:**

We conducted a retrospective cohort study using a population‐based database to investigate the epidemiology of HZ in RA patients in Taiwan during the period of 2000‐2011. A total of 27 609 newly diagnosed and eligible RA cases were identified, and 110 436 non‐RA cases were matched for age and sex at a ratio of 4:1. HZ risk increased by 2.53‐fold (*P*<0.0001) in RA patients compared with the general population. Exposure to corticosteroids (adjusted odds ratio=1.73, *P*<0.0001), adalimumab (adjusted odds ratio=1.61, *P*=0.002), and rituximab (adjusted odds ratio=2.06, *P*=0.008) was associated with an increased risk of HZ in RA patients. A significant association between the use of methotrexate or corticosteroids and HZ risk was dose‐dependent (*P*_trend_<0.0001). Elevated risk of stroke was observed in RA patients with HZ (adjusted hazard ratio=1.27, *P*=0.047), particularly in those with neurological complications (adjusted hazard ratio=1.54, *P*=0.015). A 2.30‐fold significantly increased risk of stroke within 90 days after HZ occurrence was observed in RA patients compared with those without HZ (*P*=0.02). Furthermore, death risk increased in RA patients with HZ (adjusted hazard ratio=1.18, *P*=0.026).

**Conclusions:**

The risk of HZ and HZ‐related stroke has increased in RA patients. Monitoring the occurrence of HZ in RA patients and preventing HZ‐related stroke or mortality during a specific immunosuppressive therapy are important.

**Clinical Perspective**

**What Is New?**

- Among the more than 20 million enrollees in a nationwide database, the risk of developing herpes zoster (HZ) increased 2.53‐fold in rheumatoid arthritis (RA) patients compared with the general population.
- A significantly increased risk of HZ was seen in RA patients receiving nonsteroidal anti‐inflammatory drugs combined with other immunosuppressive agents, including conventional synthetic disease‐modifying antirheumatic drugs, corticosteroids, and biologics.
- Elevated risk of stroke was observed in RA patients with HZ, particularly in those with neurological complications.
- A 2.30‐fold significantly increased risk of stroke within 90 days after HZ occurrence was observed in RA patients compared with those without HZ.

**What Are the Clinical Implications?**

- The risk of HZ and HZ‐related stroke has increased in RA patients.
- It is important to closely monitor the occurrence of HZ in RA patients during a specific immunosuppressive therapy and to immediately start efficient antiviral treatment to prevent the development of severe HZ.

## Introduction

Herpes zoster (HZ) is a significant global health burden and results from the reactivation of the varicella‐zoster virus (VZV) after a latency period following a primary infection.^1^ Approximately 50% of people living to the age of 85 years will develop HZ.^2^ Previous epidemiological studies have identified several potential risk factors associated with HZ, including aging, female sex, ethnicity, genetic susceptibility, and cellular immune dysfunction.^3^, ^4^ Complications occur in almost half of older people with HZ, including postherpetic neuralgia, ophthalmic HZ, and meningoencephalitis.^5^ Complications often affect a patient's quality of life, resulting in increased medical care costs.^6^

The Consortium of Rheumatology Researchers of North America registry data showed that VZV infection was the most frequent opportunistic infection in rheumatoid arthritis (RA) patients.^7^ Several population‐based studies in Western countries demonstrated that RA patients have an increased HZ risk compared with the general population.^8^, ^9^, ^10^ Among RA patients in Western countries, those treated with corticosteroids and/or antitumor necrosis factor (anti‐TNF) biologics appeared to be at a higher risk.^9^, ^10^, ^11^ The incidence of HZ in Asia was higher (Japan 4.15 per 1000 person‐years; Taiwan 4.89‐5.67 per 1000 person‐years) than in Western countries (United States 3.2‐3.7 per 1000 person‐years; Europe 3.7 per 1000 person‐years).^12^ However, little is known about the epidemiology of HZ in Asian RA patients, and the association between HZ and immunosuppressive agents in RA patients is controversial. In addition, several epidemiologic studies have demonstrated an increased risk of stroke after HZ.^13^, ^14^, ^15^ A recent systematic review and meta‐analysis showed that the risk of stroke is higher in patients with RA than in the general population.^16^ However, little is known about the association of HZ with stroke in RA patients. Therefore, we conducted a retrospective cohort study using a nationwide, population‐based database to investigate the epidemiology of HZ and the association of HZ complications with stroke in RA patients in Taiwan during the period 2000‐2011.

## Methods

### Data Source

The National Health Insurance Research Database (NHIRD) consists of detailed healthcare information representing more than 99% of Taiwan's total population and includes inpatient and ambulatory care claims from 1996 to 2011. The Longitudinal Health Insurance Database 2000 contains all of the original claim data of 1 000 000 individuals randomly sampled from the Registry for Beneficiaries of the NHIRD, which was released by the National Health Research Institutes. The National Health Research Institutes confirmed that the random samples were representative of the general population in Taiwan. The data from the NHIRD were deidentified forms of secondary information in an anonymous format released to the public for research purposes. This study was conducted in compliance with the Declaration of Helsinki, has been approved by the Institutional Review Board of Taichung Veterans General Hospital (CE13151B‐3), and the requirement of patient informed consent was waived. The methods were carried out in accordance with the approved guidelines.

### Definitions

In this study patients with different diseases were primarily classified using the International Classification of Diseases, Ninth Revision, Clinical Modification (ICD‐9‐CM) codes. The diagnosis of RA (ICD‐9‐CM code 714.0) was made according to the 1987 American College of Rheumatology criteria^17^ and the NHIRD's Registry of Catastrophic Illness Patient Database. The ICD‐9‐CM code for HZ was 053.0. The diagnosis of HZ was required to have been made after the RA identification index date. The complication of HZ was in accordance with a diagnosis code (Table S1).^18^ The diagnosis of HZ‐related stroke was required to have been made after the HZ identification index date and was defined by patients with a diagnosis code of stroke (ICD‐9‐CM codes 430‐438). The study end point was defined as the onset of new HZ or death during the 12‐year follow‐up period (2000‐2011).

To understand the association between comorbidities in RA patients and HZ, we chose several RA‐related comorbidities to study, including chronic obstructive pulmonary disease (ICD‐9‐CM codes 490‐492 and 496) and diabetes mellitus (ICD‐9‐CM code 250). In addition, HZ‐related comorbidities (eg, chronic kidney disease [CKD, ICD‐9‐CM code 585] and human immunodeficiency virus disease [ICD‐9‐CM codes 042‐044]) were also analyzed in this study. To investigate the association between comorbidities in RA patients with HZ and stroke, we chose several cardiovascular‐disease‐related comorbidities (including atrial fibrillation [ICD‐9‐CM code 427.31], dyslipidemia [ICD‐9‐CM code 272], and hypertension [ICD‐9‐CM codes 401‐405]) and pharmacological treatments (eg, statins) to study.

RA treatments were subdivided into 5 medication groups according to the immunosuppressive drugs used: (1) nonsteroidal anti‐inflammatory drugs (NSAIDs); (2) conventional synthetic disease‐modifying antirheumatic drugs (csDMARDs), including methotrexate (MTX), hydroxychloroquine, sulfasalazine, leflunomide, and cyclosporine; (3) corticosteroids; (4) anti‐TNF biologics, including adalimumab and etanercept; and (5) non–anti‐TNF biologics (rituximab, B‐cell depletion agent). Immunosuppressive medication exposure analysis was conducted to study the drugs‐used status before 365 days of the initial HZ diagnosis index date.^14^ Controls were assigned the index date of their corresponding HZ case patient. The corticosteroid dose was estimated based on the most recent prescription and converted to average daily prednisone equivalents: (1) low dose, <5 mg/day; (2) medium dose, 5 to <10 mg/day; and (3) high dose, ≥10 mg/day.^19^ The MTX dose was estimated based on the most recent prescription and converted to average weekly MTX equivalents: (1) low dose, <5 mg/week; (2) medium dose, 5 to <10 mg/week; and (3) high dose, ≥10 mg/week.^19^

### Study Population

**Figure 1:**
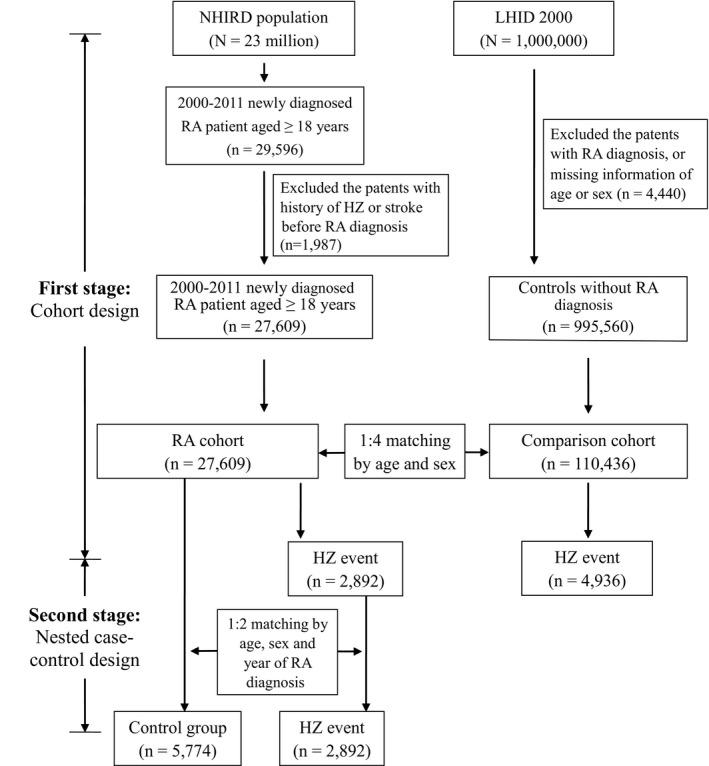
Flow chart of case selection in this study. The selection of rheumatoid arthritis (RA) patients and age‐ and sex‐matched non‐RA control subjects was from the National Health Insurance Research Database (NHIRD). HZ indicates herpes zoster; LHID 2000, the Longitudinal Health Insurance Database 2000.

We conducted a retrospective cohort study of patients ≥18 years old who were newly diagnosed with RA (29 596 cases) from January 1, 2001, to December 31, 2011, in the NHIRD. We excluded patients who had received an HZ or stroke diagnosis before the RA diagnosis as well as those who died within 180 days after RA diagnosis (1987 cases). The age‐matched non‐RA control group (age ≥18 years) was selected from the NHIRD Longitudinal Health Insurance Database 2000 and excluded cases with missing information regarding age or sex, those with a history of RA or HZ diagnosis, and those who died before July 1, 2001. Newly diagnosed RA cases (27 609 cases) and non‐RA controls (110 436 cases) were matched by age and sex using a ratio of 1:4 (Figure 1). In addition, we conducted a nested case‐control study to further determine the association of HZ risk with immunosuppressive medications in RA patients. As the control group, RA patients without HZ were matched for age, sex, and RA disease duration at the time of HZ infection with the RA‐HZ case group at a ratio of 2:1 (Figure 1, second stage). The HZ infection date was in the index date for cases and their matched controls. Comorbidities and the used medications were measured during the 365‐day period before the index date.^11^ For the study of HZ‐related stroke, the diagnosis of stroke was required to have been made after the HZ identification index date and was defined by patients with a diagnosis code of stroke. Therefore, we excluded patients who had received a stroke diagnosis before the HZ diagnosis date. HZ complications and stroke were tracked during the 365‐ and 730‐day periods after the HZ index date, separately.^18^

### Statistical Analysis

Data are presented as means±SD for continuous variables and as proportions for categorical variables. Differences between continuous values were analyzed using the independent t‐test for continuous variables and the chi‐squared test for categorical variables. The incidences of newly diagnosed HZ in RA patients and the control group were calculated. The multivariable Cox proportional hazards model was adjusted for age, sex, and comorbidity before being used to identify independent factors contributing to the development of HZ in the RA‐to‐control cohort. To quantify associations between HZ and exposure to specific immunosuppressive drugs, we used conditional logistic regression to estimate the crude and adjusted odd ratios and 95%CIs. The multivariable model was adjusted for covariates possibly associated with HZ, including age, sex, and comorbidities. All analyses were conducted using SAS statistical software version 9.3 (SAS Institute, Inc, Cary, NC). A *P*‐value of <0.05 was considered to indicate statistical significance.

## Results

### Characteristics of the Study Cohort

A total of 27 609 newly diagnosed and eligible RA cases were identified during the period of 2000‐2011. The control group was matched for age and sex at a ratio of 4:1, and a total of 110 436 non‐RA cases were selected (Figure 1). The characteristics of the enrolled participants are summarized in Table 1. Approximately 22.6% of the RA patients were older than 65 years. RA patients were predominately female (77.4%). RA patients had a significantly higher prevalence of chronic kidney disease, chronic obstructive pulmonary disease, dyslipidemia, human immunodeficiency virus infection, and hypertension than the non‐RA controls (*P*<0.0001).

**Table 1:** Baseline Characteristics (N=138 045)

Variable	RA Cohort N=27 609 (%)	Non‐RA Cohort N=110 436 (%)	*P* Value
Age, y	53.6 (14.1)	53.5 (14.2)	0.35
<45	7501 (27.2)	30 004 (27.2)	
45 to 64	13 869 (50.2)	55 476 (50.2)	
≥65	6239 (22.6)	24 956 (22.6)	
Sex			>0.99
Female	21 371 (77.4)	85 484 (77.4)	
Male	6238 (22.6)	24 952 (22.6)	
Comorbidity			
Atrial fibrillation	294 (1.1)	1235 (1.1)	0.45
Chronic kidney disease	678 (2.5)	2182 (2.0)	<0.0001^a^
COPD	9087 (32.9)	30 580 (27.7)	<0.0001^a^
Diabetes mellitus	4683 (17.0)	18 594 (16.8)	0.62
Dyslipidemia	6864 (24.9)	25 215 (22.8)	<0.0001^a^
HIV infection	12 (0.04)	17 (0.02)	0.004^a^
Hypertension	9591 (34.7)	36 134 (32.7)	<0.0001^a^
Statins used	2829 (10.3)	10 562 (9.6)	<0.0001^a^

COPD indicates chronic obstructive pulmonary disease; HIV, human immunodeficiency virus; RA, rheumatoid arthritis.

a A *P*‐value of <0.05 was indicated a significant difference in variable between RA and non‐RA cohort.

### Increased Risk of HZ in RA Patients

**Figure 2:**
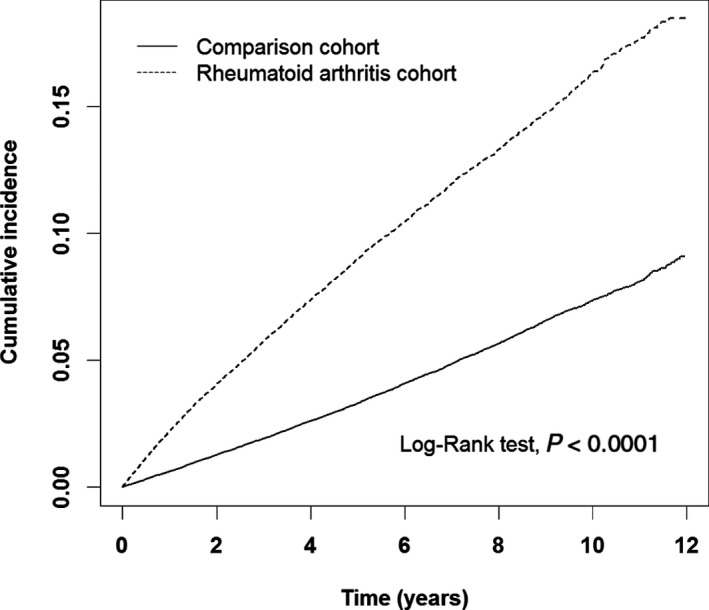
Cumulative incidence of herpes zoster (HZ) in rheumatoid arthritis (RA) patients and non‐RA comparison subjects.

Among the total of 27 609 RA patients, 2892 (10.47%) were newly diagnosed with HZ after they had been diagnosed with RA (Figure 1). The incidence rate of HZ was higher in RA patients compared with non‐RA controls (18.3 versus 7.2 per 1000 person‐years; Table 2). After multivariate analysis, including adjustment for age, sex, and comorbidities, a 2.53‐fold increased risk of developing HZ was observed in RA patients compared with the non‐RA controls (95%CI 2.42‐2.65, *P*<0.0001). Kaplan‐Meier analysis also showed that the cumulative incidence of HZ was higher in RA patients than in non‐RA subjects (*P*<0.0001, Figure 2). In addition, the risk of HZ occurrence in all age‐ and sex‐stratified RA patients was higher than that in the non‐RA cohort (*P*<0.0001, Table S2).

**Table 2:** Specific Subgroup Analysis for New‐Onset HZ (N=138 045)

Variable	Event	Person‐Y	Incidence Rate (Per 1000 Person‐Y)	Crude HR (95%CI)	*P* Value	Adjusted HR^a^ (95%CI)	*P* Value
RA							
No	4936	683 102	7.2	1.0 (reference)		1.0 (reference)	
Yes	2892	158 161	18.3	2.54 (2.42‐2.66)	<0.0001^b^	2.53 (2.42‐2.65)	<0.0001^b^
Age, y							
<45	1004	245 131	4.1	1.0 (reference)		1.0 (reference)	
45 to 64	4547	424 577	10.7	2.62 (2.45‐2.81)	<0.0001^b^	2.37 (2.21‐2.54)	<0.0001
≥65	2277	171 555	13.3	3.27 (3.03‐3.52)	<0.0001^b^	2.74 (2.53‐2.98)	<0.0001
Sex							
Female	6232	660 427	9.4	1.0 (reference)		1.0 (reference)	
Male	1596	180 836	8.8	0.94 (0.89‐0.99)	0.02^b^	0.90 (0.85‐0.95)	0.0003^b^
Atrial fibrillation							
No	7740	834 066	92.8	1.0 (reference)		1.0 (reference)	
Yes	88	7197	122	1.33 (1.08‐1.64)	0.008^b^	0.94 (0.76‐1.16)	0.54
Chronic kidney disease							
No	7658	827 437	9.3	1.0 (reference)		1.0 (reference)	
Yes	170	13 826	12.3	1.34 (1.15‐1.56)	0.0002^b^	0.92 (0.79‐1.07)	0.29
COPD							
No	4841	610 232	7.9	1.0 (reference)		1.0 (reference)	
Yes	2987	231 031	12.9	1.63 (1.56‐1.71)	<0.0001^b^	1.29 (1.23‐1.35)	<0.0001^b^
Diabetes mellitus							
No	6141	711 755	8.6	1.0 (reference)		1.0 (reference)	
Yes	1687	129 508	13.0	1.52 (1.44‐1.60)	<0.0001^b^	1.05 (0.99‐1.12)	0.10
Dyslipidemia							
No	5413	662 131	81.8	1.0 (reference)		1.0 (reference)	
Yes	2415	179 132	135.0	1.66 (1.58‐1.74)	<0.0001^b^	1.21 (1.14‐1.27)	<0.0001^b^
Hypertension							
No	4480	580 872	77.1	1.0 (reference)		1.0 (reference)	
Yes	3348	260 391	129.0	1.67 (1.60‐1.75)	<0.0001^b^	1.12 (1.06‐1.7)	<0.0001^b^
Statins used							
No	6921	777 543	89.0	1.0 (reference)		1.0 (reference)	
Yes	907	63 720	142.3	1.62 (1.51‐1.74)	<0.0001^b^	1.05 (0.97‐1.14)	0.23

COPD indicates chronic obstructive pulmonary disease; HR, hazard ratio; HZ, herpes zoster; RA, rheumatoid arthritis.

a Adjusted for age, sex, atrial fibrillation, chronic kidney disease, COPD, diabetes mellitus, dyslipidemia, and hypertension.

b A *P*‐value of <0.05 was indicated that variable was associated with herpes zoster.

### Risk of HZ in RA Patients Receiving Different Immunosuppressive Medications

To further estimate an adjusted odds ratio (aOR) for HZ in RA patients receiving different immunosuppressive medications, we conducted a nested case‐control study using conditional logistic regression. After full adjustment, no significantly increased risk of HZ was observed in RA patients receiving NSAIDs compared with nonusers (aOR=1.18, 95%CI 0.92‐1.51, *P*=0.18). However, a significantly increased risk of HZ was seen in RA patients receiving NSAIDs combined with other immunosuppressive medications, including csDMARDs, corticosteroids, and biologics (aOR=1.66, 95%CI 1.30‐2.10, *P*<0.0001). We further analyzed the risk of HZ in RA patients receiving each group of immunosuppressive agents. This showed that csDMARDs (aOR=1.30, 95%CI 1.16‐1.46, *P*<0.0001) and corticosteroids (aOR=1.73, 95%CI 1.51‐1.98, *P*<0.0001) were associated with an increased risk of HZ (Table 3). Among csDMARDs, several drugs were associated with an elevated risk of developing HZ, including hydroxychloroquine (aOR=1.15, 95%CI 1.04‐1.28, *P*=0.006), sulfasalazine (aOR=1.20, 95%CI 1.09‐1.32, *P*=0.0002), leflunomide (aOR=1.26, 95%CI 1.08‐1.46, *P*=0.003), MTX (aOR=1.31, 95%CI 1.19‐1.43, *P*<0.0001), and cyclosporine (aOR=1.44, 95%CI 1.25‐1.66, *P*<0.0001). A dose‐dependent association was observed between MTX and a greater odds ratio for HZ (*P*_trend_<0.0001). A dose‐dependent association was also found in corticosteroids (<5 mg/day, aOR=1.53, 95%CI 1.33‐1.77, *P*<0.0001; 5 to <10 mg/day, aOR=2.26, 95%CI 1.93‐2.65, *P*<0.0001), which was associated with a greater odds ratio for HZ (*P*_trend_<0.0001) after full adjustment.

**Table 3:** Odds Ratios for the Risk of HZ According to Medications Used in Rheumatoid Arthritis Patients (N=8666)

Variable	HZ Case (N=2892)	Control (N=5774)	Crude OR (95%CI)	*P* Value	Adjusted OR^a^ (95%CI)	*P* Value
NSAID (ref: NSAID nonusers)	2798 (96.7)	5555 (96.2)	1.17 (0.92‐1.50)	0.20	1.18 (0.92‐1.51)	0.18
NSAID+other immunosuppressive medications^b^ (ref: NSAID alone)	2707 (96.8)	5261 (94.7)	1.66 (1.31‐2.11)	<0.0001^c^	1.66 (1.30‐2.10)	<0.0001^c^
csDMARDs (ref: csDMARDs nonusers)	2404 (83.1)	4572 (79.2)	1.30 (1.15‐1.45)	<0.0001^c^	1.30 (1.16‐1.46)	<0.0001^c^
Methotrexate	1319 (45.6)	2278 (39.5)	1.29 (1.18‐1.41)	<0.0001^c^	1.31 (1.19‐1.43)	<0.0001^c^
<5 mg/w	730 (25.2)	1385 (24.0)	1.17 (1.05‐1.30)	0.0039^c^	1.19 (1.07‐1.33)	0.002^c^
5 to <10 mg/w	413 (14.3)	640 (11.1)	1.43 (1.25‐1.65)	<0.0001^c^	1.46 (1.27‐1.67)	<0.0001^c^
≥10 mg/w	176 (6.1)	253 (4.4)	1.55 (1.26‐1.89)	<0.0001^c^	1.57 (1.28‐1.92)	<0.0001^c^
*P*_trend_			<0.0001^c^		<0.0001^c^	
Hydroxychloroquine	806 (27.9)	1444 (25.0)	1.16 (1.05‐1.28)	0.0042^c^	1.15 (1.04‐1.28)	0.006^c^
Sulfasalazine	1955 (67.6)	3673 (63.6)	1.19 (1.09‐1.31)	0.0002^c^	1.20 (1.09‐1.32)	0.0002^c^
Leflunomide	295 (10.2)	481 (8.3)	1.25 (1.07‐1.46)	0.0041^c^	1.26 (1.08‐1.46)	0.003^c^
Cyclosporine	375 (13.0)	549 (9.5)	1.42 (1.23‐1.63)	<0.0001^c^	1.44 (1.25‐1.66)	<0.0001^c^
Corticosteroids (ref: Corticosteroid nonusers)	2583 (89.3)	4781 (82.8)	1.74 (1.51‐1.99)	<0.0001^c^	1.73 (1.51‐1.98)	<0.0001^c^
<5 mg/d	1663 (57.5)	3473 (60.1)	1.54 (1.34‐1.77)	<0.0001^c^	1.53 (1.33‐1.77)	<0.0001^c^
5 to <10 mg/d	798 (27.6)	1131 (19.6)	2.27 (1.94‐2.65)	<0.0001^c^	2.26 (1.93‐2.65)	<0.0001^c^
≥10 mg/d	122 (4.2)	177 (3.1)	2.21 (1.70‐2.88)	<0.0001^c^	2.19 (1.68‐2.85)	<0.0001^c^
*P*_trend_			<0.0001^c^		<0.0001^c^	
TNF antagonist (ref: TNF antagonist nonusers)	255 (8.8)	411 (7.1)	1.26 (1.07‐1.49)	0.0052^c^	1.27 (1.08‐1.50)	0.004^c^
Adalimumab	82 (2.8)	104 (1.8)	1.59 (1.19‐2.13)	0.0019^c^	1.61 (1.20‐2.16)	0.002^c^
Etanercept	192 (6.6)	335 (5.8)	1.15 (0.96‐1.39)	0.12	1.16 (0.97‐1.40)	0.11
Rituximab (ref: Rituximab nonusers)	27 (0.9)	27 (0.5)	2.01 (1.17‐3.43)	0.01^c^	2.06 (1.21‐3.53)	0.008^c^
Statins (ref: Statin nonusers)	345 (11.9)	665 (11.5)	1.04 (0.91‐1.20)	0.57	0.99 (0.85‐1.14)	0.87

csDMARD indicates conventional synthetic disease‐modifying antirheumatic drug; HZ, herpes zoster; NSAID, nonsteroidal anti‐inflammatory drug; OR, odds ratio; RA, rheumatoid arthritis; TNF, tumor necrosis factor.

a Adjusted for age, sex, atrial fibrillation, chronic kidney disease, chronic obstructive pulmonary disease, diabetes mellitus, dyslipidemia, and hypertension.

b Other immunosuppressive medications included were csDMARDs, corticosteroids, TNF antagonist, and rituximab.

c A *P*‐value of <0.05 was indicated that medication use was associated with herpes zoster.

Current anti‐TNF biologic users had a significantly increased risk of HZ compared with nonusers (aOR=1.27, 95%CI 1.08‐1.50, *P*=0.004). Among anti‐TNF biologic users, an increased risk of HZ in adalimumab users was detected (aOR=1.61, 95%CI 1.20‐2.16, *P*=0.002). In addition, a significantly increased risk of HZ in RA patients receiving rituximab (B‐cell depletion agent) compared with nonusers (aOR=2.06, 95%CI 1.21‐3.53, *P*=0.008) was observed.

### An Increased Risk of Stroke in RA Patients With HZ‐Related Neurological Complications

Our results showed an increased risk of hospitalization in RA patients with HZ compared with that in patients without HZ (adjusted hazard ratio [aHR] =1.36, 95%CI 1.28‐1.45, *P*<0.0001, Table S3). Moreover, an elevated risk of stroke following HZ was observed in RA patients compared with those without HZ (aHR=1.27, 95%CI 1.00‐1.60, *P*=0.047, Table 4). We further investigated the association of different HZ complications with stroke in RA patients and demonstrated a further increase of stroke risk in those with neurological complications (aHR=1.54, 95%CI 1.09‐2.19, *P*=0.015). The mortality analysis indicated an increased risk of death in RA patients with HZ compared with those without HZ (aHR=1.18, 95%CI 1.03‐1.34, *P*=0.026, Table S3).

**Table 4:** Multivariate Analysis for Risk for Stroke Among RA Patients With Different HZ Phenotypes

Variable	N	Event	Person‐Y	Incidence Rate (Per 1000 Person‐Y)	Crude HR (95%CI)	*P* Value	Adjusted HR^a^ (95%CI)	*P* Value
RA without HZ	5475	186	20 366	91.3	1.0 (reference)		1.0 (reference)	
RA with HZ	2744	116	9982	116	1.27 (1.01‐1.60)	0.04^b^	1.27 (1.00‐1.60)	0.047^b^
HZ with neurological complication	721	38	2503	152	1.66 (1.17‐2.35)	0.004^b^	1.54 (1.09‐2.19)	0.015^b^
HZ with cranial complication	185	6	625	96.1	1.05 (0.47‐2.37)	0.91	0.85 (0.38‐1.91)	0.68
HZ with other complication	229	9	700	129	1.42 (0.73‐2.78)	0.30	1.19 (0.61‐2.34)	0.60
HZ without complication	1609	63	6154	102	1.12 (0.84‐1.49)	0.44	1.20 (0.90‐1.60)	0.20

HR indicates hazard ratio; HZ, herpes zoster; RA, rheumatoid arthritis.

a Adjusted for age, sex, atrial fibrillation, chronic kidney disease, chronic obstructive pulmonary disease, diabetes mellitus, dyslipidemia, and hypertension.

b A *P*‐value of <0.05 was indicated that HZ‐related complication was associated with stroke.

Additionally, we analyzed the temporal relationship between stroke and the occurrence of the HZ infection. The results showed a 2.30‐fold significantly increased risk of stroke within 90 days after HZ occurrence in RA patients compared with those without HZ (95%CI 1.13‐4.69, *P*=0.02, Table S4).

## Discussion

Among the more than 20 million enrollees in Taiwan's NHIRD, the risk of developing HZ increased 2.53‐fold in RA patients compared with the general population. Exposure to MTX (aOR=1.31, *P*<0.0001), corticosteroids (aOR=1.73, *P*<0.0001), adalimumab (aOR=1.61, *P*=0.002), or rituximab (aOR=2.06, *P*=0.008) was associated with an increased risk of HZ. The use of corticosteroids or MTX showed a strong dose‐dependent association with HZ (*P*_trend_<0.0001). A significantly increased risk of stroke was observed in RA patients with HZ, particularly in those with neurological complications. A 2.30‐fold significantly increased risk of stroke within 90 days after HZ occurrence was observed in RA patients compared with those without HZ (*P*=0.02). In addition, the risk of hospitalization and death in RA patients with HZ was higher than in those without HZ.

A long‐term population‐based cohort study in the United States reported that the incidence of HZ has increased >4‐fold over the last 60 years.^20^ Temporal increases in the incidence of HZ have also been reported in Taiwan.^21^ Nevertheless, the cause of the temporal increase in HZ remains uncertain. The HZ incidence was higher in Taiwan (4.04‐6.24 per 1000 person‐years)^21^ than in the United States (3.2‐3.7 per 1000 person‐years)^22^ or Europe (3.7 per 1000 person‐years).^23^ In this study, we found that the incidence rate of HZ was significantly higher in RA patients compared with the general population. In addition, the risk of developing HZ increased 2.53‐fold in RA patients compared with the general population.

Our results indicated that advanced age is a major risk factor for HZ in Taiwan, which is consistent with other local data.^24^ Several population‐based studies showed that most HZ cases occurred in the ≥45‐year age group and were associated with declining cellular immunity.^1^, ^2^, ^3^, ^4^ However, we found that the HZ risk occurs earlier in RA patients than in the general population; thus, in the RA cohort, the incidence rate at <45 years of age (9.7 per 1000 person‐years, Table S2) is higher than that observed in the 45‐ to 64‐year age group in the general population (8.5 per 1000 person‐years). In addition, serious infections are a major concern in RA patients and result in increased hospitalization and mortality.^25^ Our results showed a significantly increased risk of hospitalization or death in RA patients with HZ compared with that in patients without HZ, which may be associated with RA‐related immunological dysfunction and/or comorbidities.^26^

Previous studies indicated that RA patients have an increased risk of cardiovascular disease, including cerebrovascular events.^27^, ^28^ Additionally, the prevalence of cardiovascular disease‐associated comorbidities (eg, hypertension, dyslipidemia) was higher in RA patients compared with the general population. Our results showed that RA patients had a slightly higher prevalence of dyslipidemia (24.9% versus 22.8%) and hypertension (34.7% versus 32.7%) than the non‐RA controls (*P*<0.0001), which was consistent with previous studies.^27^, ^28^ Additionally, a slightly increased risk of developing HZ was observed in patients with dyslipidemia (1.21‐fold, 95%CI 1.14‐1.27) and hypertension (1.12‐fold, 95%CI 1.06‐1.18) compared with that in the controls (*P*<0.0001). Ciccone et al^29^ demonstrated that connective tissue diseases can influence the occurrence of atherosclerosis in carotid arteries. To investigate role of HZ in the occurrence of stroke among RA patients, we further conducted a nested case‐control study using Cox proportional hazard models to examine the risks of stroke in RA‐HZ cases and RA without HZ, adjusted for several vascular risk factors (eg, diabetes mellitus, dyslipidemia, and hypertension). After adjustment, an increased risk of stroke was still revealed in RA patients with HZ. Therefore, our results showed that HZ is an independent risk factor for stroke in RA patients.

Statins are lipid‐lowering drugs that reduce the risk of cardiovascular disease. A recent study in the United Kingdom showed that statin therapy leads to a slightly elevated risk of HZ (aHR=1.13).^30^ Although our data showed that a higher proportion of statins was used in the RA cohort compared with the general population (10.3% versus 9.56%, *P*<0.0006), after full adjustment, the multivariate analysis showed that statins used did not cause significantly increased HZ risk (aHR=1.05, *P*=0.23). Additionally, further multivariate analysis among RA patients also indicated no significantly increased risk of HZ in RA patients receiving statins compared with nonusers (aHR=0.99, *P*=0.87) after full adjustment. To date, the effect of statins on HZ risk in RA patients is uncertain, and further long‐term studies are needed to confirm this hypothesis.

MTX is the most commonly used csDMARD for RA. Although MTX can prevent joint damage, concerns have been raised regarding its side effects.^31^ A systematic literature search indicated that long‐term, low‐dose use of MTX does not appear to be a risk factor for infection.^32^ However, the evidence for an increased risk of HZ in RA patients receiving MTX is controversial.^8^, ^9^, ^10^, ^11^ In addition, the MTX dose threshold of immunosuppression for HZ occurrence is uncertain. In the present study we found an elevated risk of HZ in RA patients receiving a medium or high dose of MTX (5 to <10 or ≥10 mg/week). Furthermore, our results showed that exposure to MTX significantly increased HZ risk in RA patients in a dose‐dependent manner (*P*_trend_<0.0001). MTX has been shown to suppress interferon‐γ, which plays an important role in the host's immune response to virus infection, which may cause VZV reactivation.^33^ Besides MTX, we also found that other csDMARDs (eg, hydroxychloroquine, sulfasalazine, leflunomide, and cyclosporine) were associated with an increased risk of HZ in RA patients, similar to the findings of studies in the United States and Europe.^10^, ^11^

Corticosteroids are potent immunosuppressive drugs that are widely used in the treatment of rheumatic diseases.^34^ Previous studies have shown that corticosteroid therapy is associated with the risk of serious infection in older RA patients^35^; in particular, current and recent doses have the greatest impact on infection risk.^36^ Our results showed that elevated HZ risk was positively correlated with the dose of corticosteroids, which supports the 2013 European League Against Rheumatism recommendations that clinicians consider tapering corticosteroids for RA patients in remission.^37^

Because TNF‐α plays a critical role in the control of viral infection, anti‐TNF‐α biologics may facilitate the reactivation of viral infection.^36^, ^38^ However, the association between anti‐TNF‐α biologic therapy and the risk of HZ was controversial.^8^, ^9^, ^10^, ^11^ Consistent with the findings of the Germany RA cohort study,^10^ our results showed that the use of adalimumab (monoclonal anti‐TNF‐α antibodies) was significantly associated with an increased risk of HZ compared with that in nonusers, whereas no significant association of HZ risk with the TNF‐α receptor fusion protein (etanercept) was observed. This discrepancy may be related to different characteristics of the 2 anti‐TNF‐α biologics.^39^

It is worth noting that we found a significantly increased risk of HZ in RA patients receiving therapy with rituximab, which is a monoclonal antibody targeting CD20 of B lymphocytes and affects cellular immunity.^40^ Rituximab has been approved for the treatment of RA patients who have failed to respond to 1 or more anti‐TNF‐α agents.^41^ Therefore, we thought that patients who received rituximab therapy may have a severe RA with worse cellular immunity, which may explain the increased risk of HZ in these patients.

In Taiwan RA disease is considered a catastrophic illness. Additionally, in accordance with the recommendation from the American College of Rheumatology, well‐established RA patients should receive antirheumatic therapy. Our data showed that ≈96.39% (8353/8666) of RA patients received NSAIDs during the period 2000‐2011. Therefore, we further compared the risk of HZ in RA patients receiving therapy with NSAIDs alone and NSAIDs combined with other groups of immunosuppressive agents (Table S5). After full adjustment, a significantly increased risk of HZ was observed in RA patients receiving NSAIDs combined with high‐dose (>5 mg/day) corticosteroids (aOR=1.95, 95%CI 1.38‐2.76, *P*=0.0002) or NSAIDs combined with more than 1 group of immunosuppressive agents, including csDMARDs, corticosteroids, and biologics (aOR=1.79, 95%CI 1.41‐2.28, *P*<0.0001). We thought that patients with more severe RA might be more likely to use higher doses of corticosteroids and to combine multiple immunosuppressive agents. Therefore, we estimated that the severity of RA may be a risk factor for HZ. Further studies are required to confirm this hypothesis.

In this study an increased risk of stroke was observed in RA patients with HZ after full adjustment, as compared with that in RA patients without HZ (aHR=1.27, *P*=0.047). Additionally, a further increase of stroke risk was observed in RA patients with HZ neurological complications (aHR=1.54, *P*=0.015). Furthermore, our results showed a 2.3‐fold increased risk of stroke within 90 days after HZ occurrence in RA patients compared with those without HZ (*P*=0.02), which is consistent with a recent study for HZ‐related stroke in patients with autoimmune diseases in the United States.^18^ Increasing evidence showed that VZV vasculopathy and stroke were associated with HZ.^42^, ^43^ On VZV reactivation, the virus spreads transaxonally and infects cerebral arteries and then causes neurological complications. Clinical studies found that VZV can spread to large cerebral arteries and then cause thrombosis, inflammation, and occlusion of vessels.^44^, ^45^ Inflammatory processes play a key role in stroke. Accumulation of monocytes/macrophages in the vascular wall can cause atherogenesis.^46^ A recent study^47^ found that the VZV induced upregulation of matrix metalloproteinases 1, 3, and 9 in brain vascular adventitial fibroblasts, which is associated with disruption of the vessel wall integrity and aneurysm formation. Previous studies also found that matrix metalloproteinases produced by inflammatory cells in virus‐infected arteries may further potentiate pathological vascular remodeling.^48^ During later disease stages, inflammatory cell activation can induce plaque rupture and thrombus formation, increasing stroke risk.^45^ Based on published literature, we thought that these reasons may explain why HZ‐related neurological complications such as HZ neuralgia were associated with a higher risk of stroke.

In addition, a previous study found that the VZV may penetrate to deeper tissue of immunocompromised individuals and then cause neurological complications.^49^ A recent study demonstrated that immunosuppression had a significant association with the risk of postherpetic neuralgia after HZ.^50^ Our hospital‐based study also found a high proportion (29.1%) of neurological complications in RA patients with HZ, the most common of which was HZ neuralgia.^51^ To confirm our data, we further analyzed the status of Doppler echocardiography examination in RA patients before stroke. A higher incidence rate was detected in Doppler echocardiography examination of RA patients with HZ compared with those without HZ (119 versus 101 per 1000 person‐years). Additionally, our hospital‐based data showed that a total of 7 RA patients with HZ‐related stroke had a carotid ultrasonography examination record during the period of 2013‐2016. In accordance with the records, ≈85.7% (n=6) of them had atherosclerotic plaque or increased intima‐media thickness. We hypothesized that lower cell‐mediated immunity in RA patients may lead to higher levels of virus during VZV reactivation, and thus, an increased risk of HZ‐related neurological complications caused the occurrence of stroke. Further large‐scale studies are needed to confirm this.

This study has several limitations. First, the NHIRD contains only medical claims data without laboratory data. Therefore, the examination results of risk factors for stroke (eg, echo color Doppler of carotid arteries and atherosclerotic plaques) were not available. The second limitation is the absence of individual disease activity data on RA. However, based on the British Society for Rheumatology guidelines, which specify that RA patients must have a 28‐joint disease activity score >5.1 (severe RA) before starting biological therapy,^52^ we thought that patients with more severe RA may be more likely to use biologics and higher doses of corticosteroids. Therefore, we estimated that the severity of RA may be a risk factor for HZ. Further studies are required to confirm this. The third limitation of this retrospective cohort study is a selection bias. However, the NHIRD consists of detailed healthcare information representing more than 99% of Taiwan's total population. We thought that the cohort members should have been representative of the population of all patients with RA. Hence, selection bias would have been minimized. Additionally, because zoster vaccine was available in Taiwan until October 2013, vaccination information was not available during the period of this study (2000‐2011). Finally, the NHIRD does not contain detailed information on lifestyle factors (eg, smoking, chemical exposure) or individual health status (eg, malnutrition, genetic factors) that are associated with HZ and RA.^4^

The major strength of this study is that we used a nationwide database with medical care records minimally affected by selection and recall biases. In addition, the large sample size of the NHIRD records (over 20 million enrollees, including patients and the general population) and long‐term follow‐up period (2000‐2011) enhanced the statistical power and accuracy of this study. We believe that this study provides useful information that can help increase physicians’ awareness in assessing the possibility of HZ and outcomes in patients during the period of immunosuppressive therapy.

## Conclusions

Our data showed an increased risk of HZ in RA patients receiving different immunosuppressants, particularly corticosteroids and biologics. Higher rates of HZ‐related hospitalization, stroke, and mortality were found in this study. Furthermore, a further increase of stroke risk was observed in RA patients with HZ‐related neurological complications. A significantly increased risk of stroke within 90 days after HZ occurrence was observed in RA patients compared with those without HZ. Therefore, it is important to closely monitor the occurrence of HZ in RA patients during a specific immunosuppressive therapy and to immediately start efficient antiviral treatment to prevent the development of severe HZ.

## Sources of Funding

This study was supported in part by grants from Taichung Veterans General Hospital, Taiwan (TCVGH‐1057329D, TCVGH‐105G213, TCVGH‐NHRI10505, and TCVGH‐1057308C).

## Disclosures

None.

## Supporting information


**Table S1.** ICD‐9‐CM Codes for Herpes Zoster Categories
**Table S2.** Multivariable Analysis of Baseline Factors for Rheumatoid Arthritis Patients With New‐Onset Herpes Zoster
**Table S3.** Multivariable Analysis for Risk for Hospitalization and Death Among Rheumatoid Arthritis Patients With and Without Herpes Zoster
**Table S4.** Incidence Rate of Stroke Among Rheumatoid Arthritis Patients With or Without Herpes Zoster Infection
**Table S5.** Association Between Medications Used and Herpes Zoster Among Rheumatoid Arthritis Patients (n=8353)
