# Supplementary material for: Significant Associations of Neurological Complications of Herpes Zoster With Stroke in Rheumatoid Arthritis Patients
Source: J Am Heart Assoc. 2017 Jul 19;6(7):e006304. doi: 10.1161/JAHA.117.006304 (PMC5586320; doi:10.1161/JAHA.117.006304)
Supplement: Supplementary file 1 — Table S1. ICD‐9‐CM Codes for Herpes Zoster Categories Table S2. Multivariable Analysis of Baseline Factors for Rheumatoid Arthritis Patients With New‐Onset Herpes Zoster Table S3. Multivariable Analysis for Risk for Hospitalization and Death Among Rheumatoid Arthritis Patients With and Without Herpes Zoster Table S4. Incidence Rate of Stroke Among Rheumatoid Arthritis Patients With or Without Herpes Zoster Infection Table S5. Association Between Medications Used and Herpes Zoster Among Rheumatoid Arthritis Patients (n=8353) [file JAH3-6-e006304-s001.pdf]

Supplemental Material

Table S1. ICD-9-CM codes for herpes zoster (HZ) categories

HZ subgroup	ICD-code	Description
HZ with neurological complication	053.0	Herpes zoster with meningitis
	053.10	Herpes zoster with unspecified nervous system complication
	053.12	Postherpetic trigeminal neuralgia
	053.13	Postherpetic polyneuropathy
	053.14	Herpes zoster myelitis
	053.19	Herpes zoster with other nervous system complications
HZ with cranial complication	053.11	Geniculate herpes zoster
	053.20	Herpes zoster dermatitis of eyelid
	053.21	Herpes zoster keratoconjunctivitis
	053.22	Herpes zoster iridocyclitis
	053.29	Herpes zoster with other ophthalmic complications
	053.71	Otitis externa due to herpes zoster
HZ with other complication	053.79	Herpes zoster with other specified complications
	053.8	Herpes zoster with unspecified complication
HZ without complication	053.9	Herpes zoster without complication

Table S2. Multivariable analysis of baseline factors for rheumatoid arthritis (RA) patients with new-onset herpes zoster (HZ)

Variable	RA cohort			Non-RA cohort			Adjusted HR†	*P* value
	Event	person-years	Incidence rate*	Event	person-years	Incidence rate*	(95% CI)	
Overall	2,892	158,161	18.3	4,936	683,102	7.2	2.53(2.42–2.65)	**<0.0001**
Age group								
<45	463	47,786	9.7	541	197,344	2.7	3.43 (3.03–3.88)	**<0.0001**
45-64	1,620	80,503	20.1	2,927	344,074	8.5	2.33 (2.19–2.48)	**<0.0001**
≥65	809	29,872	27.1	1,468	141,684	10.4	2.56 (2.35–2.79)	**<0.0001**
Sex								
Female	2,275	124,890	18.2	3,957	535,537	7.4	2.48 (2.36–2.62)	**<0.0001**
Male	617	33,270	18.5	979	147,566	6.6	2.81 (2.54–3.11)	**<0.0001**

*per 1,000 person-years.

†Adjusted for age, sex, atrial fibrillation, chronic kidney disease, chronic obstructive pulmonary disease, diabetes mellitus, dyslipidemia and hypertension.

Table S3. Multivariable analysis for risk for hospitalization and death among rheumatoid arthritis (RA) patients with and without herpes zoster (HZ)

Variable	Event	person-years	Incidence rate (per 1,000 person-years)	Crude HR (95% CI)	*P* value	Adjusted HR* (95% CI)	*P* value
**Hospitalization**							
RA without HZ	2,557	14,677	174	1.0 (reference)		1.0 (reference)	
RA with HZ	1,535	6,343	242	1.36 (1.28-1.45)	**<0.0001**	1.36 (1.28–1.45)	**<0.0001**
**Mortality**							
RA without HZ	584	21,566	27.1	1.0 (reference)		1.0 (reference)	
RA with HZ	338	10,696	31.6	1.17 (1.02–1.33)	**<0.024**	1.18 (1.03–1.34)	**0.026**

*Adjusted for age, sex, atrial fibrillation, chronic kidney disease, chronic obstructive pulmonary disease, diabetes mellitus, dyslipidemia and hypertension.

Table S4. Incidence rate of stroke among rheumatoid arthritis (RA) patients with or without herpes zoster (HZ) infection

	RA without HZ infection			RA with HZ infection			Adjusted HR†	*P* value
	Event	person-years	Incidence rate*	Event	person-years	Incidence rate*	(95% CI)	
**0-90 day**	14	1,336	105.0	17	672	253.0	2.30 (1.13–4.69)	**0.02**
**91-365 day**	31	3,655	84.8	17	1,818	93.5	1.05 (0.58–1.90)	0.87
**366-730 day**	41	4,065	101.0	24	2,006	120.0	1.16 (0.70–1.92)	0.56
**>730 day**	100	11,310	88.4	58	5,487	106.0	1.18 (0.86–1.64)	0.31

*per 1,000 person-years.

†Adjusted for age, sex, atrial fibrillation, chronic kidney disease, chronic obstructive pulmonary disease, diabetes mellitus, dyslipidemia and hypertension.

Table S5. Association between medications used and herpes zoster (HZ) among rheumatoid arthritis patients (n= 8,353)

Variable	HZ case N = 2,798	Control N = 5,555	Crude OR (95% CI)	*P* value	Adjusted OR* (95% CI)	*P* value
NSAID alone	91 (3.3)	294 (5.3)	reference		reference	
NSAID + csDMARDs	173 (6.2)	559 (10.1)	1.00 (0.75-1.34)	0.99	1.00 (0.75-1.34)	0.99
NSAID + corticosteroids ≤ 5 mg/day	254 (9.1)	619 (11.1)	1.33 (1.01-1.75)	**0.04**	1.31 (0.99-1.73)	0.05
NSAID + corticosteroids > 5 mg/day	95 (3.4)	156 (2.8)	1.97 (1.39-2.78)	**0.0001**	1.95 (1.38-2.76)	**0.0002**
NSAID + more than one group of immunosuppressive agents†	2185 (78.1)	3927 (70.7)	1.80 (1.41-2.29)	**<0.0001**	1.79 (1.41-2.28)	**<0.0001**

*Adjusted for age, sex, atrial fibrillation, chronic kidney disease, chronic obstructive pulmonary disease, diabetes mellitus dyslipidemia and hypertension.

†Including conventional synthetic disease-modifying anti-rheumatic drugs (csDMARDs), corticosteroids, and biologics.
